# Synovial Sarcoma: A Clinical Review

**DOI:** 10.3390/curroncol28030177

**Published:** 2021-05-19

**Authors:** Aaron M. Gazendam, Snezana Popovic, Sohaib Munir, Naveen Parasu, David Wilson, Michelle Ghert

**Affiliations:** 1 Department of Surgery, Division of Orthopaedic Surgery, McMaster University, Hamilton, ON L8V 1C3, Canada; wilsondaj@gmail.com (D.W.); ghertm@mcmaster.ca (M.G.); 2 Department of Pathology and Molecular Medicine, McMaster University, Hamilton, ON L8S 4L8, Canada; popovics@hhsc.ca; 3 Department of Radiology, McMaster University, Hamilton, ON L8V 1C3, Canada; munirsoh@hhsc.ca (S.M.); parasun@hhsc.ca (N.P.)

**Keywords:** synovial sarcoma, soft tissue sarcoma, clinical review, current concepts

## Abstract

Synovial sarcomas (SS) represent a unique subset of soft tissue sarcomas (STS) and account for 5–10% of all STS. Synovial sarcoma differs from other STS by the relatively young age at diagnosis and clinical presentation. Synovial sarcomas have unique genomic characteristics and are driven by a pathognomonic t(X;18) chromosomal translocation and subsequent formation of the SS18:SSX fusion oncogenes. Similar to other STS, diagnosis can be obtained from a combination of history, physical examination, magnetic resonance imaging, biopsy and subsequent pathology, immunohistochemistry and molecular analysis. Increasing size, age and tumor grade have been demonstrated to be negative predictive factors for both local disease recurrence and metastasis. Wide surgical excision remains the standard of care for definitive treatment with adjuvant radiation utilized for larger and deeper lesions. There remains controversy surrounding the role of chemotherapy in the treatment of SS and there appears to be survival benefit in certain populations. As the understanding of the molecular and immunologic characteristics of SS evolve, several potential systematic therapies have been proposed.

## 1. Introduction

Synovial sarcoma is a relatively rare malignancy representing a soft tissue sarcoma (STS) of uncertain differentiation. It accounts for 5–10% of all STS [1,2,3]. The age-adjusted incidence is 0.81/1,000,000 in children and 1.42/1,000,000 in adults with approximately 1000 patients diagnosed with synovial sarcoma in the United States each year [4]. Synovial sarcoma is unique from other STS as it presents at a younger mean age of onset and commonly occurs in adolescents and young adults (mean age of 39 years at diagnosis) and affects both sexes equally [5]. Synovial sarcoma is the most common non-rhabdomyosarcoma STS in children, representing 30% of STS diagnosed in childhood [4,6].

## 2. Clinical Presentation

### 2.1. Location

Although many synovial sarcomas originate near articular structures, the name synovial sarcoma is a misnomer in that these lesions do not originate from intra-articular synovium, but from primitive mesenchymal cells [7]. Synovial sarcomas most commonly present as soft tissue masses but cases of primary synovial sarcoma of bone have been reported [8]. These lesions can occur anywhere in the body, with the majority arising in the extremities, particularly in the lower extremity in the anatomic structures adjacent to the knee joint [4,9]. Synovial sarcomas are considered the most common STS of the foot [6,10,11].

### 2.2. Signs and Symptoms

Synovial sarcomas often do not present with the typical STS presentation of a large and quickly growing painless mass [9]. Instead, the majority of synovial sarcomas are slow growing and the mean duration of symptoms before diagnosis is approximately 2 years [12]. In comparison to other STS, the duration of symptoms are long and patients may have pain or joint contractures that precede swelling [13]. Only half of patients have clinical findings consistent with STS according to the National Institute for Health and Care Excellence (NICE) guidelines [12,13]. Given the insidious onset, younger age at presentation and atypical presenting symptoms, these patients may be initially clinically misdiagnosed with benign processes including myositis, synovitis, bursitis or tendonitis.

## 3. Imaging

### 3.1. Plain Radiographs

**Figure 1:**
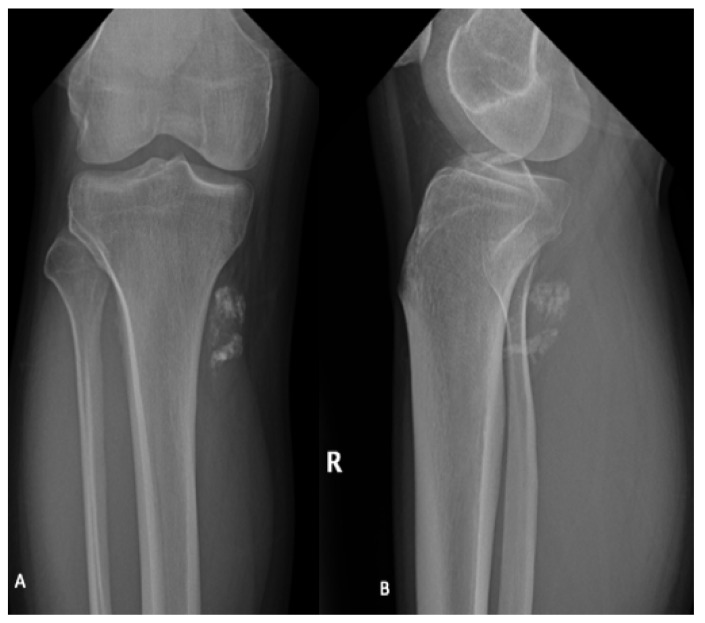
AP (**A**) and lateral (**B**) radiographs of the right (R) knee and lower leg in a 31-year-old male demonstrate coarse calcifications within the soft tissues adjacent to the posteromedial tibial plateau, corresponding to a biopsy proven synovial sarcoma. No significant articular abnormality. Adjacent bony structures appear unremarkable.

Plain radiographs are not required for diagnosis but are typically performed as part of the initial workup and can identify adjacent bony remodeling, bone invasion or calcification of the soft tissue mass (Figure 1) [14,15,16]. Typically, synovial sarcoma presents as a well-defined or lobulated soft tissue mass on plain radiographs. Punctate calcifications, particularly around the periphery of the lesion, are visualized in one third of patients [17]. Occasionally, more extensive calcification can be visualized and can mimic bone forming tumors including osteosarcoma and myositis ossificans [17].

### 3.2. Cross-Sectional Imaging

**Figure 2:**
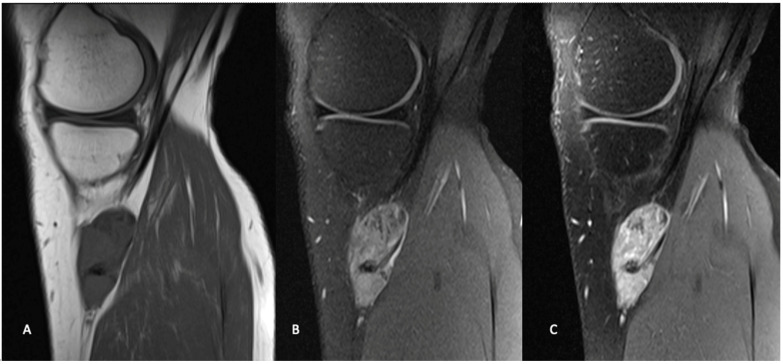
Sagittal MR images in the same patient demonstrate a periarticular soft tissue mass situated posteromedial to the proximal tibia, in close relation to the pes anserine tendons. The mass demonstrates intermediate signal on T1 weighted images (**A**), and heterogeneously high signal on T2 weighted fat-saturated images (**B**). T1 weighted fat-saturated after gadolinium administration (**C**), demonstrates heterogeneously avid enhancement. Known areas of calcification are low signal on all sequences (arrow).

Similar to other STS, magnetic resonance imaging (MRI) with and without contrast is the gold standard for diagnostic imaging for synovial sarcoma (Figure 2) [17]. MRI defines the local extent of the soft tissue mass and surrounding edema and provides excellent visualization of the mass with respect to the surrounding anatomy, which is critical for preoperative planning. The utilization of gadolinium contrast can differentiate between hemorrhagic or necrotic areas and areas of solid viable tumor. As with most STS, synovial sarcomas are typically heterogenous with low intensity on T1 and high intensity on T2-weighted images with post-gadolinium enhancement [17].

Although synovial sarcomas generally present as a non-specific heterogenous mass, there are some unique features, which can aid in differentiation from other STS. Synovial sarcomas predominately present as well-defined, heterogeneously enhancing solid tumors that are multilobulated in nature [17]. A triple signal intensity demonstrating areas of hyperintensity, isointensity and hypointensity indicating the mix of cystic and hemorrhagic areas, cellular elements and fibrotic areas can be characteristic [17]. Smaller tumors, particularly those smaller than 5 cm in diameter, often show homogeneous enhancement which can be mistaken for a benign process [18]. Several findings on MRI have been found to be predictive of high-grade lesions including the absence of calcifications and presence of hemorrhage and the triple signal intensity [19].

Computed tomography (CT) with contrast can be utilized when an MRI is contraindicated or unavailable. Synovial sarcoma appears hypointense compared to muscle with heterogeneity in larger lesions [20]. CT allows for better visualization of soft-tissue calcifications and local bone reaction [17].

## 4. Diagnosis and Staging

### 4.1. Biopsy

A biopsy and pathologic assessment are required to differentiate synovial sarcoma from other STS subtypes and define the tumor grade. As with all STS, a biopsy should be performed prior to definitive surgery to avoid inadequate resection and misdiagnosis [21]. Options for biopsy include incisional biopsies, core needle biopsies and fine needle aspirations (FNA).

Historically, open incisional biopsies have been considered the gold standard for soft tissue lesions as they provide larger volumes of tissue. When compared to CNB and FNA, IB tend to have higher diagnostic accuracy but this comes with a higher rates of complications when compared to percutaneous techniques [22]. Core needle biopsies retrieve more tissue than FNA and have higher diagnostic accuracy [23]. Fine needle aspirates are rarely used in STS due to the small quantity of sample material obtained and a limited ability to assess lesional architecture [22]. Advances in diagnostic imaging has allowed for image guided percutaneous biopsies which has improved the diagnostic accuracy of these techniques [24]. Given the lower morbidity and relatively high diagnostic accuracy of CNB, image guided CNB are the preferred method of biopsy, particularly for deeper tumors. When open biopsies are performed, the biopsy principles must be observed to reduce biopsy-related complications [25].

### 4.2. Staging

Staging investigations are imperative and allow for a better understanding of disease prognosis and risk of recurrence or metastases. The tumor stage also helps to formulate a treatment plan by a multidisciplinary sarcoma team. Staging of synovial sarcoma involves cross-sectional imaging of the affected extremity, systematic staging with a chest CT, and pathologic assessment. The two most commonly utilized staging systems are the American Joint Committee on Cancer (AJCC) system and the Enneking staging system [26,27]. The Enneking staging system has remained largely unchanged since its introduction in 1980 while the AJCC system has evolved significantly and is currently on its Eighth edition. The Enneking staging system relies on tumor grade, local extent of disease and presence of metastases. The AJCC staging system is based on anatomical site of primary tumor and tumor size (pT) tumor grade, nodal involvement (N), and presence of metastases (M) [27].

Synovial sarcomas are malignant and metastasize, most commonly to the lungs with up to 13% of patients having distant metastases at the time of diagnosis [5]. Historically, it was thought that synovial sarcoma had a predilection for metastases to lymph nodes, necessitating the need for further advanced imaging and possible sentinel node biopsy [28]. However, these findings were based on small case series and more recent Surveillance, Epidemiology and End Results (SEER) database findings suggest that the rates of lymph node metastases are in line with other STS and do not require additional workup beyond thorough physical examination [29].

## 5. Diagnostic and Molecular Pathology

### 5.1. Pathology

**Figure 3:**
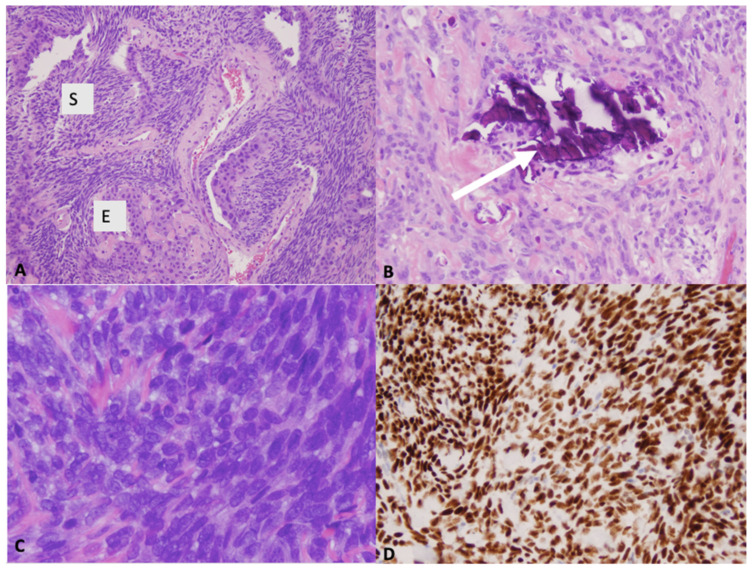
(**A**) Microscopic images showing biphasic tumor composed of spindle cell (S) and epithelioid (E) component; H&E ×100 and (**B**) occasional calcifications (arrow); H&E ×200). (**C**) Microscopic image showing poorly differentiated morphology (H&E ×400). (**D**) Immunohistochemistry demonstrating diffuse and strong nuclear staining for the transcriptional corepressor TLE1 (×200).

The gross pathology appearance of synovial sarcoma is usually tan or grey and may be multinodular or multicystic [7]. Most synovial sarcomas are 3–10 cm in diameter. Smaller lesions (<1 cm) can occur in hands and feet [3,30]. Histologically, synovial sarcoma is a monomorphic spindle cell sarcoma with variable epithelial differentiation (Figure 3) [3]. It presents as one of three variants: monophasic, biphasic or poorly differentiated. In the monophasic variant, the tissue is comprised entirely of spindle-cells whereas in biphasic synovial sarcoma, there are epithelial and spindle-cell components present [7]. In one third of synovial sarcomas, areas of calcifications and/or ossification can be found. In both monophasic and biphasic variants, there may be poorly differentiated areas with increased cellularity, greater nuclear atypia and high mitotic activity (6 mitoses/mm^2^ >10 mitoses per 1.7 mm^2^) [3]. Occasionally, the entire tumor shows poorly differentiated morphology. On immunohistochemistry, diffuse expression of bcl-2 is typically seen. In 60% of cases, these tumours stain positive for CD99 [3]. Immunohistochemistry also demonstrates strong and diffuse nuclear staining for the transcriptional corepressor TLE1 found in the large majority of synovial sarcomas [3]. NY-ESO-1 is also expressed strongly in most synovial sarcomas and can help differentiate it from other spindle cell neoplasms [31].

### 5.2. Molecular Pathology

**Figure 4:**
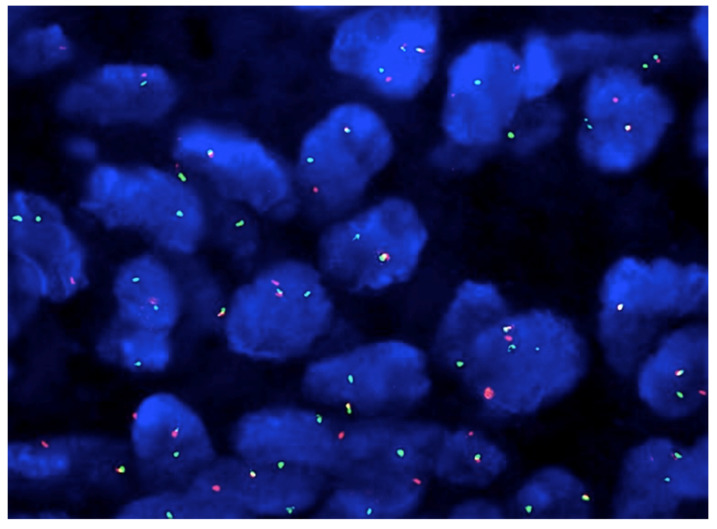
Molecular study using fluorescence in situ hybridization (FISH) break apart probe confirmed a diagnosis of synovial sarcoma detecting the SS18 gene arrangement (split red and green signal) (×200).

Synovial sarcoma is characterized by a pathognomonic translocation t(X:18) which is present in >95% of cases (Figure 4) [32]. This translocation leads to the expression of different SS18:SSX oncogenic fusion proteins, which drive sarcomagenesis. Subtypes include SS18:SSX1 and SS18:SSX2 and less commonly SS18:SSX4 [33]. Both fluorescence in situ hybridization (FISH) and reverse transcription polymerase chain reaction (RT-PCR) testing have been validated in the diagnosis of this translocation [34]. Almost all SS18-SSX2 synovial sarcomas show monophasic morphology and are significantly more common in women. Rare cases are associated with t(X;20) and SS18L1-SSX1 fusion transcript [3].

## 6. Treatment

As with all sarcomas, the treatment plan for synovial sarcoma is individualized to each patient. Both patient and tumor variables are taken into account in a multidisciplinary setting to determine the ideal treatment strategy for each patient.

### 6.1. Surgical Management

The mainstay of treatment for synovial sarcoma remains surgical excision with negative margins with the addition of radiotherapy and/or chemotherapy based on patient and tumour characteristics [21]. Historically, patients were often treated with amputation but advances in adjuvant therapy and cross-sectional imaging have allowed the majority of patients to be treated with limb-salvage surgery [35].

The goal of limb-salvage surgery in synovial sarcoma is to achieve oncologic control of the tumour while providing the patient with a functional limb postoperatively. Negative surgical margins are of upmost importance as they predict both local recurrence and overall survival [36,37,38]. While no specific guidelines exist regarding ideal negative margins in synovial sarcoma, surgical management is similar to that of other STS. For superficial tumors or small (<5 cm) deep tumors not intimately associated with critical structures, a wide excision with negative margins (1–2 cm) alone could be considered sufficient [39]. In tumours that are closely associated with neurovascular structures or bone, the epineurium, adventitia or periosteum is utilized as the margin to allow for a functional limb postoperatively [38,40]. In these cases, very close or microscopically positive margins may occur and radiotherapy is essential to decrease local recurrence risk [41,42]. Carefully planned microscopically positive margins on a fixed structure (bone, nerve, vessel) have been shown to not increase the risk for local recurrence in setting of neoadjuvant radiotherapy [42]. Given the heterogeneity and lack of standardization in the literature, recommendations regarding margin management must be taken into context of the individual patient. Both tumor and anatomic factors should be taken into consideration when determining acceptable surgical margins [43].

Due to its atypical presentation (slow-growing, painful mass), synovial sarcomas have a high rate of presentation following an unplanned excision, with up to 50% of patients presenting after unplanned excision [12,44]. Unplanned excisions of synovial sarcomas result in high rates of residual disease, particularly for larger and deeper tumors and increased risk for local recurrence even after re-excision [44,45]. In the case of referral following unplanned excision, patients should be re-staged, and the original histology reviewed at a sarcoma referral center. Tumor bed excision should be performed in patients with residual disease with the goal of complete tumor resection and negative margins [46]. Typically, these resections are extensive in nature given that areas of potential contamination must be removed, with may necessitate reconstructive procedures [45]. There is a paucity of data to guide the utilization of radiotherapy in this population; however, radiotherapy is recommended as it would be in a primary presentation [46].

In select cases, limb-salvage techniques are not recommended and primary amputation may be required [35]. Amputation is considered in patients with tumour location that necessitates excision of vital structures which would result in poor limb function [35]. Patients who present following an unplanned excision may require amputation if there is extensive contamination of vital structures or major joints [35]. Finally, older patients or those with extensive medical comorbidities may not be able to tolerate a major operation and amputation can be considered [35].

### 6.2. Radiation Therapy

Neoadjuvant or adjuvant radiation therapy is recommended for larger tumors (>5 cm), or in any case where a close margin may be required to preserve a major neurovascular structure or bone [21]. In large registry database studies, radiotherapy has been shown to improve local control and may have overall survival benefit in patients with synovial sarcoma [47,48,49]. Radiotherapy can be administered pre or postoperatively with differing protocols. Preoperative radiation is associated with higher wound complication rates whereas postoperative radiation can case fibrosis and joint stiffness which may lead to worse long term functional outcomes [50]. Regardless of timing, intensity-modulated radiation therapy (IMRT) is becoming the preferred method of radiation delivery in patients with STS [21,51]. Intensity-modulated radiation therapy allows for a higher dose of radiation to more closely contour the tumor, which reduces the volume of radiation to the surrounding normal tissues. The utilization of preoperative IMRT for STS has been shown to reduce wound complications and need for reconstructive soft-tissue flaps [52]. Radiation therapy may be considered in isolation in patients with multiple medical comorbidities or patients with metastatic disease where the risks of surgery outweigh the potential benefits [53].

### 6.3. Systemic Therapies

Unlike the majority of STS, synovial sarcoma appears to be more chemosensitive, although there is still controversy surrounding which subgroups of patients benefit from systemic therapy [54]. In general, chemotherapy is reserved for patients with high-risk tumors or advanced disease and is thought to be more effective in younger patients [55,56].

In children and adolescents with intermediate or high-risk tumours (i.e., >5 cm, nodal involvement, positive margins), adjuvant or neoadjuvant chemotherapy is generally undertaken with the most common agents being ifosfamide and doxorubicin. In the absence of available RCTs, prospective multicentred cohort studies have demonstrated adequate response to chemotherapy [57]. Recent data has demonstrated that pediatric or adolescent patients with low risk tumors (Grade 2 or Grade 3 < 5 cm) can be successfully treated with surgical intervention without systemic therapy [39].

The role of chemotherapy in adult patients with synovial sarcoma is less clear. Eilber et al. (2007) demonstrated that chemotherapy improved distant relapse-free survival in patients with high-risk synovial sarcoma [54]. Their group also published a synovial sarcoma specific nomogram that supported survival benefit of ifosfamide-based chemotherapy for certain adult patient populations [55]. Similarly, pooled data from 15 trials on advanced STS demonstrated significantly better response to chemotherapy and survival rates when compared to other STS [56]. Contrary to this, the French Sarcoma Group recently demonstrated no overall survival benefit with neoadjuvant or adjuvant chemotherapy in adult patients with synovial sarcoma [58]. However, this study included patients with low-risk tumor characteristics in which chemotherapy is unlikely to be of benefit.

Chemotherapy is also considered in metastatic or unresectable disease [56,59]. In general, anthracycline-based chemotherapy is first line for advanced STS and the addition of ifosfamide is dependent on the subtype of STS. Ifosfamide has well documented efficacy in synovial sarcoma in the palliative setting and should be considered in patients who undergo chemotherapy if the toxicities can be tolerated [59,60]. Spurrell et al. demonstrated median survival of 22 months in patients with advanced disease treated with a combination of doxorubicin and ifosfamide which was superior to either agent given in isolation [59].

### 6.4. Novel Agents

There has been interest in the development of targeted medical therapies in the treatment of synovial sarcoma [61]. New agents including receptor tyrosine kinase inhibitors, epigenetic modifiers and immunotherapies have been investigated in clinical trials. Thus far, only pazopanib, a receptor tyrosine kinase inhibitor is approved for clinical use. Pazopanib has been investigated in patients with advanced disease and has demonstrated improved progression free survival in a Phase III trial [59,62]. There has been recent advances in cell-based therapies targeting the cancer testis antigen, NY-ESO-1. Early work examining the utility of adoptive T-cell therapy with autologous T cells that have been engineered to expressive NY-ESO-1, have been promising in patients with metastatic disease [63]. However, although several larger agents have demonstrated preclinical success, clinical trials are needed to determine the role of these novel agents in the treatment of synovial sarcoma.

## 7. Prognosis

The current literature suggests the five-year survival rates ranges from 59–75% [4,5,9,62,64]. There has been a trend for improved survival over time with early cohorts from the 1960s quoting a 25–51% five-year survival rate. Local and metastatic relapse of soft-tissue sarcomas generally occur in the first two years following treatment and thus surveillance and follow-up is most intensive in this period [65]. However, synovial sarcoma is unique in this regard in that it tends to recur much later. Krieg et al. (2011) demonstrated that local recurrence occurred after a mean of 3.6 years (range 0.5–15 years) and metastases occurred at a mean of 5.7 years (range 0.5–16.3 years) [66].

There are several well documented key prognostic factors including tumor size, grade and anatomical location, patient age at diagnosis, negative surgical margins and adjuvant radiotherapy [38,55,64,67,68,69]. Osseous or neurovascular invasion, adult age, large tumour size and unplanned excision have been linked to worse prognosis [64,67,68,69,70]. The role of the subtype of oncogene protein is conflicting and does not appear to have a definite impact on outcomes [32,71,72,73]. Recent data suggests that histologic subtype appears to be prognostic, with the biphasic subtype demonstrating the highest survival rates at both five and ten years [73]. Synovial sarcomas with >20% poorly differentiated areas show more aggressive behaviour. The best outcomes are seen with tumors with histologic features <6 mitoses/mm^2^ and no necrosis [3,74].

Similar to other STS, tumor size and grade has repeatedly been shown to have prognostic value in patients with synovial sarcoma [48,54,67]. In a cohort of 1189 patients, Naing et al. (2014) demonstrated that size predicted worse overall survival [48]. Tumor location has been also been demonstrated to be of predictive value, with non-extremity based synovial sarcomas tending to have worse overall survival [5,75]. However, this may be in part due to the lack of early symptoms and later stage of presentation.

Although synovial sarcoma has a similar clinical presentation in children and adults, there is a growing evidence that they have different outcomes, with children having significantly better survival rates [4,9,39]. Utilizing registry data, Sultan et al. (2009) demonstrated the five-year survival rate for children and adolescents to be 83% compared to 62% in adults [4]. Similarly, Smolle et al. (2019) demonstrated an 89% five-year cancer specific survival rate in children compared to 75% in adults [9]. Vlenterie et al. (2015) demonstrated a clear stepwise reduction in survival with age, regardless of tumor site, size and treatment [68].

## 8. Conclusions

Synovial sarcomas (SS) represent a unique subset of STS and account for 5–10% of all STS. Synovial sarcoma differs from other STS by the relatively young age at diagnosis, anatomic location (peri-articular) and clinical presentation (slow-growing, painful lesions). Given this unique presentation, it is important for orthopaedic surgeons to recognize synovial sarcoma and avoid inappropriate interventions. Synovial sarcomas have unique genomic characteristics and are driven by a pathognomonic t(X;18) chromosomal translocation and subsequent formation of the SS18:SSX fusion oncogenes. Surgical excision remains the mainstay of treatment with radiation therapy utilized in high-risk tumors. Chemotherapy appears to have benefit in high-risk tumors in younger patients but there remains conflicting data in the adult population. Synovial sarcoma requires longer follow-up due to its risk of late recurrence.
